# The Ventilation of Bed Rooms

**Published:** 1881-12

**Authors:** 


### THE VENTILATION OF BED ROOMS.

If a man were deliberately to shut himself for some six or eight hours daily in a stuffy room, with closed doors and windows (the doors not being opened even to change the air during the period of incarceration), and were then to complain of headache and debility, he would justly be told that his own want of intelligent foresight was the cause of his suffering. Nevertheless, this is what the great mass of people do every night of their lives with no thought of their imprudence. There are few bedrooms in which it is perfectly safe to pass the night without something more than ordinary precautions to secure an inflow of fresh air. Every sleeping apartment should, of course, have a fire-place with an open chimney, and in cold weather it is well if the grate contains a small fire, at least enough to create an upcast current to carry off the vitiated air of the room. In all such cases, however, when a fire is used, it is necessary to see that the air drawn into the room comes from the outside of the house. By an easy mistake it is possible to place the occupant of a bedroom with a fire in a closed house in a direct current of foul air drawn from all parts of the establishment. Summer and winter, with or without the use of fires, it is well to have a free ingress for pure air. This should be the ventilator's first concern. Foul air will find an exit if pure air is admitted in sufficient quantity, but it is not certain that pure air will be drawn away. So far as sleeping-rooms are concerned it is wise to let in air from without. The aim must be to accomplish the object without causing a great fall of temperature or a draught. The windows may be drawn down an inch or two at the top with advantage, and a fold of muslin will form a " ventilator " to take off the feeling of draught. This, with an open fire-place, will generally suffice, and produce no unpleasant consequences even when the weather is cold. It is, however, essential that the air outside should be pure. Little is likely to be gained by letting in a fog or even a town mist. —*Lancet.*

